# Lima LV, Pavinati G, Moroskoski M, Gil NLM, Takenami IO, Magnabosco GT. Inequalities in the provision of rapid HIV testing in primary health care: ecological study, Paraná, 2013-2023. Epidemiol Serv Saude. 2026:35;e20250981

**DOI:** 10.1590/S2237-96222026v35e20250981.b

**Published:** 2026-10-12

**Authors:** João Paulo Cola

**Affiliations:** 1 Secretaria de Estado da Saúde do Espírito Santo, Vitória, ES, Brazil Secretaria de Estado da Saúde do Espírito Santo Vitória ES Brazil

## First round comments

Prezados autores,

Agradecemos a submissão do manuscrito RESS-2025-0981 intitulado “Desigualdades na oferta de testagem rápida do HIV na atenção primária à saúde no Paraná: estudo de séries temporais e múltiplos grupos (2013-2023)” à Revista Epidemiologia e Serviços de Saúde: revista do SUS (RESS).

Após a revisão do seu artigo, identificamos a necessidade de uma revisão maior para que o manuscrito esteja de acordo com os padrões editoriais da revista e para viabilizar sua publicação.

Além dos pontos detalhados pelos revisores, é importante que revisem os seguintes pontos maiores:

1: Revisar o termo utilizado como “oferta de testagem rápido do HIV”. O estudo não mediu a oferta do teste rápido, e sim a realização do teste. Sugestão e modificar para “realização do teste rápido para HIV”.

2: Rever o termo “comportamentos espaciais” no objetivo do estudo. Sugestão em utilizar “distribuição espacial”.

3: Solicito, por gentileza, que sejam revisados os termos potencialmente estigmatizantes, especialmente aqueles relacionados ao HIV. Sempre que possível, priorize o uso de “pessoas vivendo com HIV” em vez de expressões como “diagnóstico de HIV”, “teste de HIV positivo” ou similares, de modo a alinhar o manuscrito a uma linguagem não discriminatória e centrada na pessoa.

4: Organizar a estrutura da metodologia pelo guia RECORD (https://www.equator-network.org/reporting-guidelines/record/)

5: Revise a descrição dos resultados para apresentar os achados numéricos de forma direta e precisa. Evite expressões como “cerca de” ou “aproximadamente”. Por exemplo, substituir “totalizaram, juntas, cerca de 0,03%” por “totalizaram 0,03%”.

6: Revise os s resultados para garantir que todas as análises apresentadas estejam previamente descritas na metodologia. Observa-se, por exemplo, que os autores apresentam médias aritméticas das taxas de testes realizados, mas essa análise não está explicitada na seção de métodos. É importante incluir essa informação para manter a coerência entre metodologia e resultados.

7: Padronizar os termos de análise de tendência em todo o texto, utilizando-os sempre conforme descrito na metodologia. Sugere-se empregar, preferencialmente, os termos “crescente”, “decrescente” e “estacionária”.

8: Rever o primeiro parágrafo da discussão. Os autores afirmam haver avanços na descentralização da testagem rápida para HIV, porém esse aspecto não foi mensurado no estudo. Mantenham a discussão alinhada ao objetivo do trabalho, enfatizando a análise de tendência, a distribuição espacial e os fatores contextuais relacionados aos achados.

9: Revisem o resumo para que apresente claramente os resultados numéricos. Informem as variações observadas com seus respectivos intervalos de confiança, assim como as razões de taxas e respectivos ICs obtidos nas análises de regressão binomial. Na conclusão, respondam de forma direta ao objetivo do estudo: qual foi a tendência temporal identificada? Como se deu a distribuição espacial? E quais fatores estiveram associados?

Também é desejável que revisem os seguintes pontos menores:

Introdução:

10: (P2, L32) Descreva claramente o que seria “meta global 95-95-95”. Quais são essas metas?

11: (P2, L56) Rever a sentença “a descentralização da prevenção ao HIV a partir da oferta de testagem rápida pela APS”. A testagem de HIV é uma medida de prevenção? ou diagnostico precoce? Caso necessário, argumente como a testagem pode ser uma ação de prevenção.

Metodologia:

12: (P3, L33) Acerte censo do IBGE de 2022.

13: (P4, L20-21) Acrescentar a informação “portanto, a residência da pessoa pode ser diferente do município onde o procedimento foi realizado” nas limitações do estudo e retirar da metodologia.

14: (P5, L9-12) Essa afirmação necessita de referência.

15: (P5, L41) Valores devem ser apresentados na seção resultados.

16: (P5, L49-50) Descrever como as variáveis independentes foram padronizadas.

17: (P6, L3-4) Descrever como foi avaliado a multicolineariedade.

Resultados:

18: Na descrição dos resultados da regressão binomial, apresente somente as taxas com intervalos de confiança, retirar valor p.

19: Na tabela 1, acrescente uma coluna com a classificação da tendencia “crescente”, “decrescente” e “estacionária”.

20: Na tabela 2, acrescente as análises múltiplas.

21: Na figura 2, identifique em outra cor os municípios com testagem zerada.

22: A figura 1 está com baixa qualidade. Enviar figura com alta resolução, conforme orientações aos autores.

Discussão:

23: (P8, L30-36) Acrescente que as testagens são realizadas simultaneamente para HIV e sífilis e que a tendência crescente de testagem para HIV em determinados locais pode ter contribuído para o aumento da detecção de sífilis.

24: (P8, L38-44) Descreva melhor o paragrafo, deixando claro para o leitor de que metas estão se referindo.

25: Finalize o texto com o parágrafo de conclusão (P9, L7-13). Reorganizem o parágrafo de limitações do estudo (P9, L14-21) para que seja apresentado antes da conclusão ou, alternativamente, como segundo parágrafo da discussão.

26: Revise os links das referências. Alguns não estão funcionados.

Solicitamos que todos os comentários da revisão por pares sejam respondidos pontualmente por meio de uma carta resposta detalhada, explicando as modificações realizadas ou justificando, quando necessário, a manutenção do texto original. Para agilizar o processamento do manuscrito revisado, seja o mais específico possível em sua resposta ao(s) revisor(es) e editor. Destaque as alterações no texto do manuscrito em amarelo.

Solicito, por gentileza, que a versão revisada do manuscrito seja conferida à luz das normas de formatação da revista, com atenção especial todos os ajustes de normas e formato apontados pelos revisores. Isso garantirá que o texto esteja plenamente adequado ao padrão editorial antes do reenvio.

A nova versão do manuscrito será reconsiderada após essa reformulação e poderá passar por nova rodada de avaliação. Destacamos que esta decisão não garante a aceitação final do manuscrito.

Atenciosamente,

Dr. João Paulo Cola

Editor Associado da Revista Epidemiologia e Serviços de Saúde: revista do SUS (RESS)

Recommendation: Major revision

## Second round comments

Prezados autores,

Agradecemos a submissão da nova versão do manuscrito RESS-0981 intitulado “Desigualdades na realização de teste rápido do HIV na atenção primária: estudo de séries temporais e múltiplos grupos, Paraná, 2013-2023” à Revista Epidemiologia e Serviços de Saúde: revista do SUS (RESS).

Após uma nova revisão do seu manuscrito e avaliação das respostas aos revisores, identificamos a necessidade de uma revisão menor para que o manuscrito esteja de acordo com os padrões editoriais da revista e para viabilizar sua publicação. Solicito a revisão dos seguintes pontos:

1- P2, L4652: ainda não ficou claro como a descentralização do teste rápido é uma ação preventiva para ampliação do acesso. Sugiro suprimir a palavra “preventiva”. Ex.: Como decorrência da maior proximidade domiciliar e da possibilidade de estabelecimento de vínculo, a descentralização da testagem rápida do HIV à APS se constitui como uma estratégia de grande potencial na ampliação do acesso pela população adscrita ao diagnóstico precoce e ao tratamento oportuno do HIV, fortalecendo as práticas no âmbito do modelo de prevenção combinada.

2- P3, L22: informe que é o censo do IBGE.

3- P4, L56: os resultados numéricos de estratificação/inclusão/exclusão de amostras/população devem ser apresentados nos resultados. Sugestão que mantenha a sentença “Por fim, para modelar os fatores associados à realização de teste rápido para HIV na APS, consideraram-se apenas os municípios que tiveram, pelo menos, um procedimento registrado no SIA/SUS, no período de 2013 a 2023”, contudo nos resultados (P6, L3-10) apresente os dados numéricos da amostra/população analisada.

4- P5, L48-55: Revise as casas decimais das frequências relativas.

5- Tabela 2: Acrescente o p-valor das análises.

Solicitamos que todos os comentários da revisão sejam respondidos pontualmente por meio de uma carta resposta detalhada, explicando as modificações realizadas ou justificando, quando necessário, a manutenção do texto original. Para agilizar o processamento do manuscrito revisado, seja o mais específico possível em sua resposta ao editor. Destaque as alterações no texto do manuscrito em amarelo.

Solicito, por gentileza, que a versão revisada do manuscrito seja conferida à luz das normas de formatação da revista, com atenção especial todos os ajustes de normas e formato apontados. Isso garantirá que o texto esteja plenamente adequado ao padrão editorial antes do reenvio.

A nova versão do manuscrito será reconsiderada após essa reformulação e poderá passar por nova rodada de avaliação. Destacamos que esta decisão não garante a aceitação final do manuscrito.

Atenciosamente,

Dr. João Paulo Cola

Editor Associado da Revista Epidemiologia e Serviços de Saúde: revista do SUS (RESS) Second round comments

**Recommendation:** Minor revision

